# Guardians at the Gate: Immune System in Gastrointestinal Diseases

**DOI:** 10.3390/ijms25115933

**Published:** 2024-05-29

**Authors:** Elena Layunta, Jose Emilio Mesonero, Eva Latorre

**Affiliations:** 1 Departamento de Anatomía, Embriología y Genética Animal, Facultad de Veterinaria, Universidad de Zaragoza, 50013 Zaragoza, Spain; 2 Instituto de Investigación Sanitaria de Aragón (IIS Aragón), 50009 Zaragoza, Spain; 3 Instituto Agroalimentario de Aragón-IA2, Universidad de Zaragoza-CITA, 50013 Zaragoza, Spain; 4 Departamento de Farmacología, Fisiología y Medicina Legal y Forense, Facultad de Veterinaria, Universidad de Zaragoza, 50013 Zaragoza, Spain; 5 Departamento de Bioquímica y Biología Molecular y Celular, Facultad de Ciencias, Universidad de Zaragoza, 50009 Zaragoza, Spain

The immune system plays a key role in gastrointestinal (GI) pathologies, being responsible for protecting the body against infection, maintaining homeostasis, and regulating the inflammatory response in the GI tract. The improper or inaccurate functioning of the immune system is a critical element in GI diseases, constituting one of the most substantial challenges to global public health due to their dramatic prevalence worldwide [1]. This group of pathologies includes inflammatory bowel disease (IBD), irritable bowel syndrome (IBS), and diverticular disease (DD), as well as GI infections triggered by pathogens [2,3]. In this context, recent analysis has unveiled a notable surge in the global burden of IBD over recent decades, particularly in developing regions undergoing rapid industrialization [4], whereas the prevalence of IBS remains considerable, affecting up to 15% of the global population [5]. Moreover, DD presents a significant incidence with a prevalence of approximately 4.3% among adults aged 40 years and older in the United States [6] and an annual increase of approximately 3.5% in across Western nations [7]. GI infections caused by pathogens cause 1.6 million fatalities annually worldwide according to the World Health Organization (WHO) [8]. Even with the increased importance of GI pathologies being recognized in global healthcare, its prevalence seems to be underestimated due to challenges in diagnosis [9]. IBD relies on a combination of clinical evaluation, endoscopic findings, histopathology, and imaging studies. However, the clinical presentation of IBD can vary widely, with symptoms such as abdominal pain, diarrhea, rectal bleeding, and weight loss overlapping with those of other GI conditions, including IBS and GI infection. This diagnostic ambiguity often leads to delays in diagnosis and the misclassification of patients [10], so there is an urgent need to facilitate diagnosis. Recent studies propose the use of urine biomarkers for the diagnosis of IBD, providing a non-invasive approach [11].

The GI tract is a critical interface between the external environment and the internal milieu, working not only in digestion, secretion, motility, and nutrient absorption, but also as a gateway for potential harmful environmental elements including pathogens or dietary antigens. The complex interplay among the intestinal barrier, immunity, and beneficial gut microbiota plays a pivotal role in maintaining this delicate balance within the GI tract, named intestinal homeostasis. This equilibrium is crucial for optimal GI function, while also preventing chronic inflammation. However, disruptions in intestinal homeostasis can lead to a range of intestinal pathologies [12].

The beneficial gut microbiota comprises trillions of microorganisms, including bacteria, viruses, fungi, and archaea, which coexist symbiotically within the GI tract [13]. These microorganisms are critical for all intestinal functions [14], including protection against pathogens [15]. The immune system maintains a symbiotic relationship with this microbiota, promoting tolerance to beneficial microorganisms and controlling pathogen overgrowth. The imbalance in microbial composition, called dysbiosis, has been linked to various pathologies such as IBD, IBS, DD, and several GI infections [2]. In this context, restoring microbial balance through probiotics, prebiotics, and dietary interventions is a fascinating and promising area of research [16].

The immune system in the gut includes both innate and adaptive mechanisms. Innate immunity involves physical barriers, antimicrobial peptides, and pattern recognition receptors (PRRs) such as Toll-like receptors (TLRs), which recognize microbial components and initiate immediate defense responses [17]. Adaptive immunity, involving B and T lymphocytes, provides long-term immunity and immunological memory, ensuring a precise response to repeated exposure to pathogens [18]. Emerging evidence suggests that alterations in immunity play a crucial role in the pathogenesis of GI disease due to its importance in intestinal defense [19]. In this context, the alteration of cytokines such as TNF-α may play a key role in the pathogenesis of not only IBD and IBS [20], but also of DD, and could potentially be a target for future therapies [21].

The intestinal mucosal barrier is composed firstly by a thick mucus layer and, secondly, by a single layer of epithelial cells tightly connected by junctional complexes [22]. This barrier prevents the translocation of pathogens and harmful substances from the gut lumen into systemic circulation [23]. The mucus layer, primarily composed of mucins secreted by goblet cells, acts as a physical barrier, trapping microbes and facilitating their clearance [24]. Alterations in the intestinal mucosal barrier lead to conditions such as leaky gut syndrome, which is characterized by increased intestinal permeability and is a potential contributor not only to various GI pathologies, but also to systemic conditions including metabolic syndrome, neurodegenerative diseases, or autoimmune disorders. In fact, autoimmune pathology treatments such as dimethyl fumarate could not only improve psoriasis or multiple sclerosis, but may also be a new potential therapy in the context of inflammatory and immune-mediated intestinal diseases [25].

Recent research has elucidated the mechanisms underlying leaky gut syndrome, including the dysregulation of tight junction proteins, mucosal inflammation, and dysbiosis of the gut microbiota [26], yet the external agents triggering leaky gut syndrome are not completely understood. Several studies show that environmental toxins such as arsenic can be an important modulator of gut-mucosa-associated immune responses and gastrointestinal permeability [27], opening up potential approaches to restore intestinal homeostasis.

The immune system must differentiate between its own and foreign antigens; failures in this process can lead to autoimmune responses. Regarding the GI tract, this can manifest in several diseases such as celiac disease, where the ingestion of gluten triggers an abnormal immune response that damages the intestinal barrier of the small intestine. Recent data associate the leaky gut with autoimmune diseases. In fact, higher levels of circulating autoantibodies are found in human subjects with increased intestinal permeability, suggesting the use of positive linear correlations between serum occludin/zonulin antibodies and circulating autoantibodies in autoimmune disease diagnosis [28].

The intestinal immune system, which includes the physical barrier of the intestinal epithelium, resident immune cells (such as lymphocytes and macrophages), and antibody production (especially IgA), acts to identify and neutralize pathogens such as bacteria, viruses, and parasites. This first line of defense is crucial to prevent infections that can cause acute digestive diseases. In response to infection or tissue damage, the immune system initiates an inflammatory response to eliminate the pathogen and repair the affected tissue. However, excessive or chronic inflammation can damage intestinal tissue and contribute to GI diseases. In these conditions, inflammation persists even in the absence of clear pathogens, suggesting an autoimmune component or dysfunction in the regulation of the immune response. 

The immune system contributes to the delicate balance between protection against pathogens and tolerance to food antigens and commensal microbiota. Immune dysfunction can lead to a variety of GI disorders, from acute infections to chronic diseases such as IBD, IBS, or DD. A thorough understanding of the interactions between the immune system and the GI tract is essential for the development of new therapies and management strategies to improve digestive health. Continued research in this area promises to reveal more complex underlying mechanisms and potential therapeutic interventions, offering hope for a better quality of life for those suffering from these conditions.
